# Spontaneous Formation of Ultrasmall Unilamellar Vesicles in Mixtures of an Amphiphilic Drug and a Phospholipid

**DOI:** 10.1021/acs.langmuir.3c01023

**Published:** 2023-08-02

**Authors:** Vahid Forooqi Motlaq, Lars Gedda, Katarina Edwards, James Doutch, L. Magnus Bergström

**Affiliations:** † Department of Medicinal Chemistry, Uppsala University, P.O. Box 547, 751 23 Uppsala, Sweden; ‡ Department of Chemistry—Ångström, P.O. Box 573, Uppsala University, 751 23 Uppsala, Sweden; § ISIS Neutron and Muon Source, STFC, Rutherford Appleton Laboratory, Harwell Campus, Didcot OX11 0QX, Oxon, United Kingdom

## Abstract

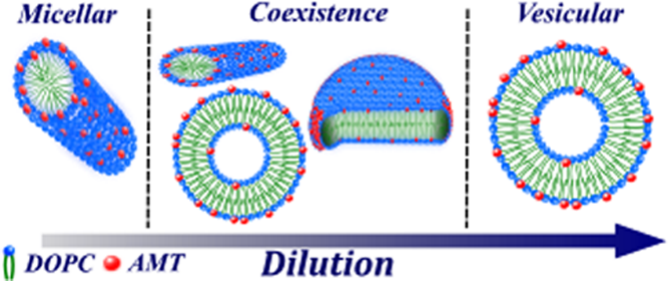

We have observed ultrasmall unilamellar vesicles, with diameters of less than 20 nm, in mixtures of the tricyclic antidepressant drug amitriptyline hydrochloride (AMT) and the unsaturated zwitterionic phospholipid 1,2-dioleoyl-sn-glycero-3-phosphocholine (DOPC) in physiological saline solution. The size and shape of spontaneously formed self-assembled aggregates have been characterized using complementary techniques, i.e., small-angle neutron and X-ray scattering (SANS and SAXS) and cryo-transmission electron microscopy (cryo-TEM). We observe rodlike mixed micelles in more concentrated samples that grow considerably in length upon dilution, and a transition from micelles to vesicles is observed as the concentration approaches the critical micelle concentration of AMT. Unlike the micelles, the spontaneously formed vesicles decrease in size with each step of dilution, and ultrasmall unilamellar vesicles, with diameters as small as about 15 nm, were observed at the lowest concentrations. The spontaneously formed ultrasmall unilamellar vesicles maintain their size for as long we have investigated them (i.e., several months). To the best of our knowledge, such small vesicles have never before been reported to form spontaneously in a biocompatible phospholipid-based system. Most interestingly, the size of the vesicles was observed to be strongly dependent on the chemical structure of the phospholipid, and in mixtures of AMT and the phospholipid 1,2-dimyristoyl-sn-glycero-3-phosphocholine (DMPC), the vesicles were observed to be considerably larger in size. The self-assembly behavior in the phospholipid–drug surfactant system in many ways resembles the formation of equilibrium micelles and vesicles in mixed anionic/cationic surfactant systems.

## 1 Introduction

Amphiphilic molecules like surfactants and phospholipids self-assemble in aqueous solutions above a certain critical aggregation concentration. While conventional surfactants usually form rather small thermodynamically stable micelles, phospholipids self-assemble to form large bilayer structures. Phospholipid bilayers, frequently mixed with other components like cholesterol, may form geometrically closed uni- or multilamellar structures called liposomes or vesicles.^1^ Vesicles occur naturally in the human body, where they may perform a variety of different intracellular and extracellular functions.^2^ Vesicles also make up important structural entities in a diverse range of areas and applications, for instance, as a structural component in enveloped viruses and in various pharmaceutical formulations and drug delivery systems.^3−5^ The size of vesicles is expected to influence several important properties, such as solubilization capacity, transportation in an extracellular matrix, ability to interact with and permeate biological membranes, spreading viral diseases, etc.

Unilamellar vesicles are classified into three main groups based on the vesicle size: small unilamellar vesicles (SUV) have a diameter in the range of 20–100 nm, large unilamellar vesicles (LUV) range between 100 nm and 1 μm, and giant unilamellar vesicles (GUV) with a size range of 1–200 μm.^6^ Liposomes are usually not thermodynamically stable structures but must be formed by, for instance, high-energy sonic fragmentation and extrusion methods. The metastable liposomes are frequently seen to grow in size with time into larger bilayer structures, indicating large bilayer aggregates to be the equilibrium structure.

Mixing two surfactants or a micelle-forming surfactant and a phospholipid may lead to the spontaneous formation of unilamellar vesicles.^7^ Spontaneously formed vesicles have been reported to form in mixtures of conventional surfactants or bile salts and phospholipids,^8−10^ as well as in mixtures of oppositely charged surfactants.^11,12^ The latter case is interesting, since the unilamellar vesicles that are formed seem to be thermodynamically stable equilibrium structures. The size of the vesicles also seems to depend on the chemical structure of the constituent surfactant molecules, and occasionally, the unilamellar vesicles have been observed to be conspicuously small in size, that is smaller than about 30 nm in diameter, sometimes even smaller than 20 nm.^13,14^

There has been a controversy over the last 30 years or so about whether unilamellar vesicles are thermodynamically stable structures or not. It seems as if a wide agreement prevails that small unilamellar vesicles formed spontaneously from two oppositely charged surfactants are indeed thermodynamically stable. On the other hand, unilamellar vesicles formed by phospholipids, and in phospholipid/surfactant mixtures, have hitherto been considered nonequilibrium structures.

In the present article, we report the observation of conspicuously small vesicles formed spontaneously by simply diluting solutions of mixed micelles in mixtures of the phospholipid 1,2-dioleoyl-sn-glycero-3-phosphocholine (DOPC) and the drug amitriptyline hydrochloride (AMT). AMT is an amphiphilic drug that self-assembles on its own into small oblate spheroidal micelles above a well-defined critical micelle concentration. The aggregation numbers of AMT micelles have been determined to be in the range of 35–45 in pure water and 0.15 M NaCl.^15^

In a recent study, we investigated the dissolution of a supported DOPC bilayer by AMT using a combination of neutron reflectivity and quartz crystal microbalance with dissipation. We were able to conclude that the AMT eventually dissolved the DOPC in mixed micelles in a two-step process, where the outer phospholipid monolayer was dissolved prior to the inner layer.^16^

## 2 Materials and Methods

### 2.1 Materials

**Figure 1:**
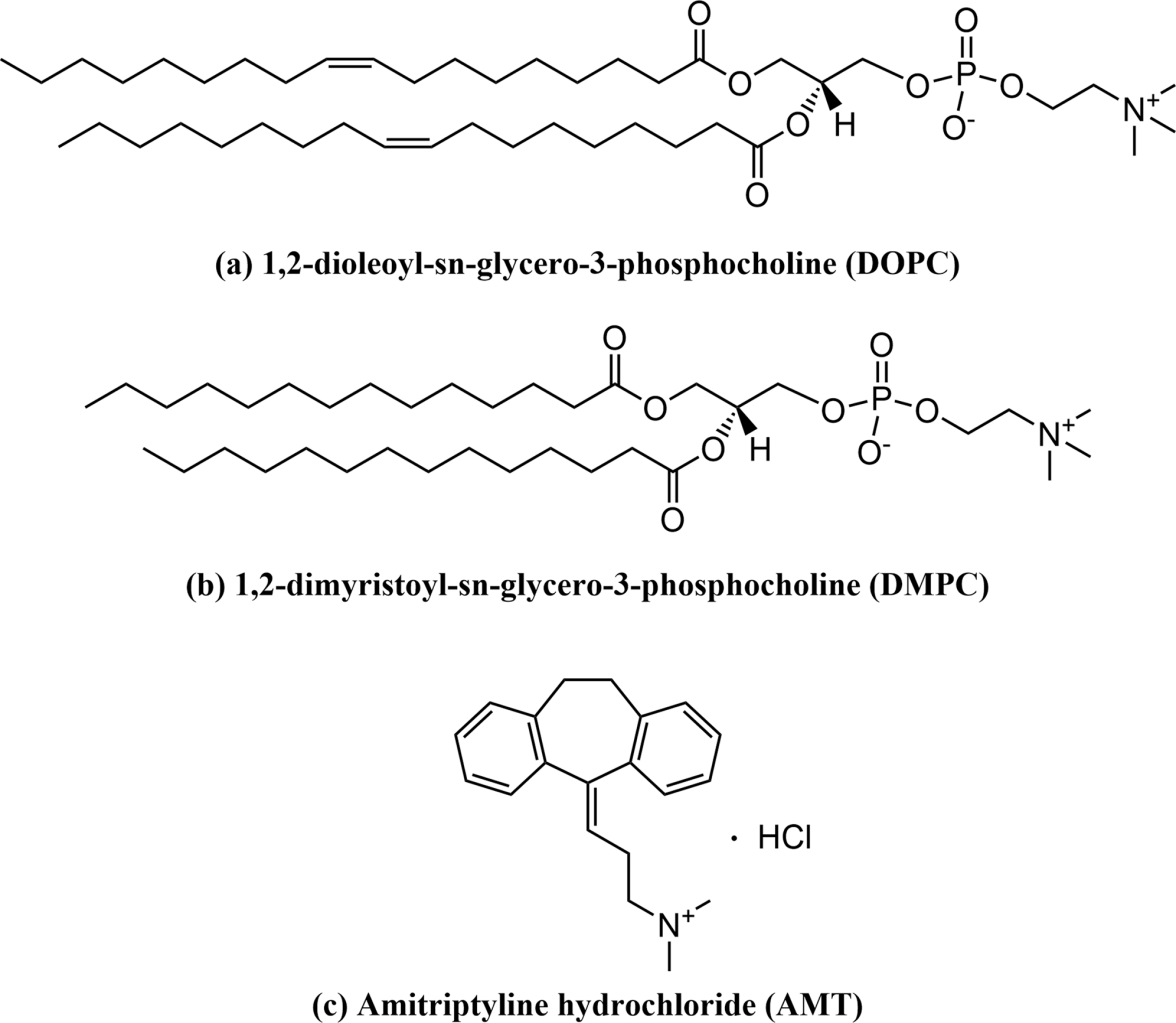
Chemical structures of (a) 1,2-dioleoyl-sn-glycero-3-phosphocholine (DOPC), (b) 1,2-dimyristoyl-sn-glycero-3-phosphocholine (DMPC), and (c) amitriptyline hydrochloride (AMT).

Amitriptyline hydrochloride (AMT) was purchased from Sigma-Aldrich, and 1,2-dioleoyl-sn-glycero-3-phosphocholine (DOPC) and 1,2-dimyristoyl-sn-glycero-3-phosphocholine (DMPC) were from Larodan AB, Solna, Sweden. Both compounds were used as received without further purification. The chemical structures of AMT and DOPC are shown in Figure 1.

The samples were prepared by simple mixing of the two components with the molar ratios of *X*_PL_ = [DOPC]/([DOPC] + [AMT]) of 0.20 and 0.25 and a total concentration of *c*_t_ = [DOPC] + [AMT] of 40 mM in 0.154 M NaCl solutions. The samples were simply mixed by magnet stirring overnight without using any external forces, i.e., ultrasound, extrusion, etc. To keep the conditions constant for all techniques, all samples were prepared in deuterium oxide. Subsequent to overnight stirring, the stock solutions were further diluted to the rest of the series’ total concentrations ranging from [DOPC] + [AMT] = 40 to 5 mM. All samples were left for equilibration at 21 °C for at least 48 h and were transparent at the time of measurement.

The critical micelle concentration of AMT was determined to be CMC = 17 mM in 0.154 M NaCl solutions in Milli-Q water from surface tension measurements using the Wilhelmy plate method. The critical aggregation concentration of DOPC is about 1–10 μM.^17^

### 2.2 Small-Angle X-ray Scattering (SAXS)

The SAXS measurements were performed on a Xeuss 2.0 Q-Xoom system, (Xenocs, Grenoble, France) equipped with a GENIX 3D Cu Ultra Low Divergence (λ = 1.54 Å) X-ray source and a two-dimensional PILATUS 3R 300K X-ray Detector (Dectris, Switzerland). The data were collected at 250 mm sample–detector distance using a low-noise flow cell (Xenocs) to cover the range of scattering vectors, i.e., 0.017–1.1 Å^–1^.

Scattering 2D images were reduced to 1D curves, normalized to an absolute scale, and the buffer and cell scatterings were subtracted from the sample scattering using the Foxtrot software package.^18^

### 2.3 Small-Angle Neutron Scattering (SANS)

Small-angle neutron scattering (SANS) experiments were performed on a time-of-flight ZOOM instrument at the ISIS neutron and muon spallation source at Rutherford Appleton Laboratory, Didcot, UK.^19^ The incident beam had a wavelength range of 1.75–16.5 Å, and the sample-to-detector distance was 4 m, giving scattering vector moduli *Q* ranging from 0.0042 to 0.84 Å^–1^. The resolution in *Q* ranges from 2.5 to 21% from low to high *Q*-values.

The samples were kept in quartz cells (Hellma) with a path length of 2 mm. The raw spectra were corrected for background from the solvent, sample cell, and other sources by conventional procedures.^20^ The obtained 2D images were reduced to 1D, normalized to the detector response, sample transmission, spectral distribution of the incident neutron beam, and wavelength distribution, and put on an absolute scale before subtraction of solvent scattering using Mantid software.^21^ The SANS data on an absolute scale (in unit cm^–1^) was normalized by means of dividing with the solute concentration in [g cm^–3^], giving the normalized intensity in unit [cm^2^ g^–1^]. The temperature was maintained at 21 °C during the measurements.

### 2.4 Data Analysis of SANS and SAXS Data

The SANS and SAXS curves were fitted with various geometrical models to find the best possible match utilizing an in-house least-squares fitting program developed by Pedersen et al.^22^ The quality of the model fits was compared using the reduced chi-square parameter as a measure. Since our measurements were carried out at a comparatively low aggregate concentration in a high electrolyte concentration (154 mM NaCl), all our data could be fitted with an appropriate form factor, and no structure factor was implemented into the fitting routine.

Because of the relatively high electron density of phosphorus in the phospholipid head groups, the scattering density of the head group is much higher than the scattering density of the hydrocarbon tail. Hence, all form factors used to fit our SAXS data take into account the core–shell structure of the aggregates. SANS data, on the other hand, could be fitted with models assuming homogeneous aggregates. Due to the limited *q*-range of our SAXS instrument, and the low contrast between aggregates and the solvent, it was not possible to acquire the reasonable quality of our SAXS data for samples with a total concentration of less than about 30 mM.

SAXS and SANS data for the samples with compositions *X*_PL_ = 0.20 and 0.25 at concentrations well above the CMC of AMT, i.e., 40 and 30 mM, were best fitted with a model for polydisperse rodlike micelles with an elliptical cross section. SANS data for most samples with concentrations of 20 mM and below were fitted with a model for coexisting disks and unilamellar vesicles. Exceptions were the samples with *X*_PL_ = 0.25 and 20 mM, which were fitted with a model for coexisting rodlike micelles, bilayer disks, and unilamellar bilayer vesicles, and the two samples with the lowest total concentration in each series (5 mM), which were fitted with a model for polydisperse unilamellar vesicles. Corrections for instrumental smearing of SANS data appeared to have a very small impact on the fitting results and were not explicitly taken into account in data analysis. More details are provided in the Supporting Information.

### 2.5 Cryo-Transmission Electron Microscopy (cryo-TEM)

The method used for cryo-TEM analysis is described earlier by Almgren et al.^23^ Briefly, samples were equilibrated at 21 °C (37 °C for DMPC) and high relative humidity within a climate chamber. A small drop of each sample (∼1 μL) was deposited on a carbon-sputtered copper grid covered with a perforated polymer film. Excess liquid was thereafter removed by blotting with a filter paper. This leaves a thin film of the solution on the grid. The sample was immediately vitrified in liquid ethane and transferred to the microscope, continuously kept below −160 °C, and protected against atmospheric conditions. Analyses were performed with a Zeiss Libra 120 transmission electron microscope (Carl Zeiss AG, Oberkochen, Germany) operating at 80 kV and in zero-loss bright-field mode. Digital images were recorded under low-dose conditions with a BioVision Pro-SM Slow Scan CCD camera (Proscan Elektronische Systeme GmbH, Scheuring, Germany).

## 3 Results and Discussion

**Figure 2:**
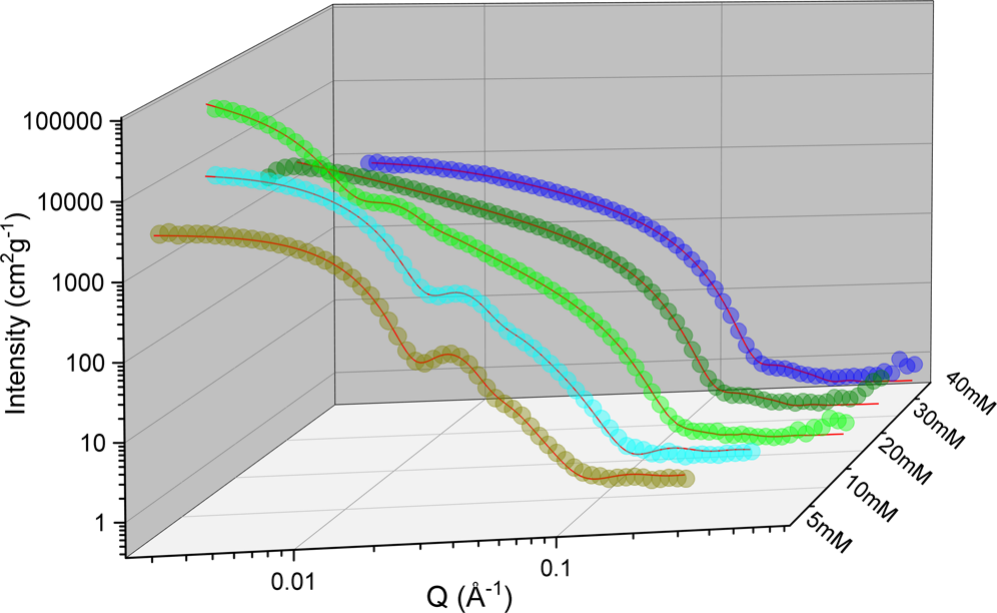
SANS profiles for samples with DOPC mole fraction *X*_PL_ = 0.25 and total concentrations [AMT] + [DOPC] = 40, 30, 20 10, and 5 mM. The scattering intensities are set to an absolute scale and normalized with respect to total concentrations.

Figure 2 shows the normalized SANS intensity as a function of scattering vector modulus *Q* for mixtures with *X*_PL_ = 0.25 and total concentrations in the range of 5–40 mM. We observe two distinct regions, a micellar region at high concentrations and a bilayer vesicular region at low concentrations. In between, there is a rather narrow regime where micelles coexist with bilayer vesicles and disks. The structural transformations upon diluting our samples can be seen in the normalized SANS data in Figure 2, as the intensity increases as micelles formed at 40 and 30 mM increase in size with decreasing concentration. Micelles transform to comparatively large bilayer structures at 20 mM, and, consequently, the SANS intensity significantly increases in magnitude. Upon further dilution of the samples, the scattering intensity decreases in magnitude as the vesicles decrease in size. The presence of vesicles in our samples can be seen as a typical oscillation appears in the SANS curves somewhere between *Q* = 0.01 and 0.1 Å^–1^. The oscillation is shifted toward lower *Q*-values as the size of the vesicles is decreased.

### 3.1 Micellar Region

#### 3.1.1 Small-Angle Neutron Scattering

The results from our model fitting analysis of samples in the micellar region, i.e., *X*_PL_ = 0.20 and 0.25 at 30 and 40 mM, are summarized in Table S1 in the Supporting Information. The micelles were found to be rodlike at *X*_PL_ = 0.25 and 40 mM, with a length equal to 17 nm and an elliptical cross section with half axes equal to *a* = 1.8 nm and *b* = 2.7 nm. The value of the minor half-axis (corresponding to about the half-thickness of the micelles) is consistent with previous reports of the chain lengths of DOPC.^24^ The micelles grow in length to 63 nm upon dilution to *c*_t_ = 30 mM, while the cross-sectional dimensions of the rodlike micelles remain unchanged. The length of rodlike micelles formed at *X*_PL_ = 0.20 is considerably larger than that in the *X*_PL_ = 0.25 series, but the cross-sectional dimensions were the same for both compositions.

#### 3.1.2 Small-Angle X-ray Scattering

**Figure 3:**
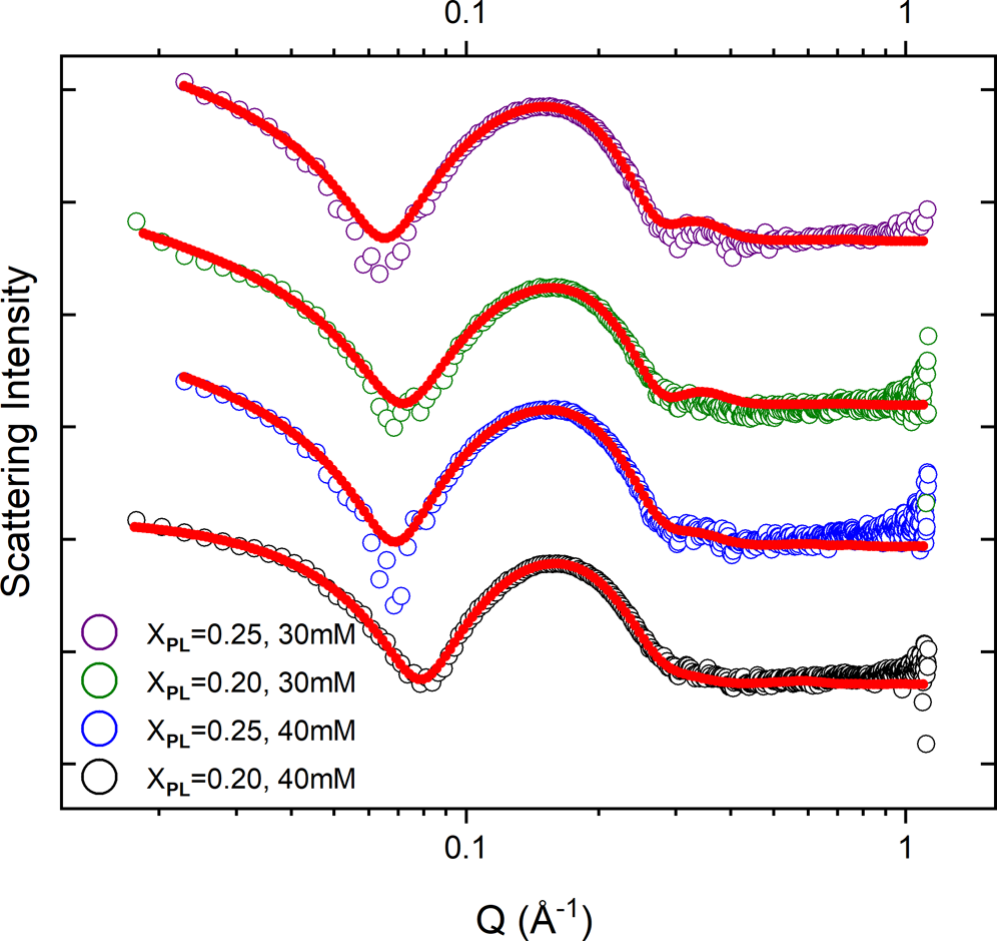
SAXS profiles for the samples with DOPC mole fractions *X*_PL_ = 0.20 and 0.25 and total concentrations [AMT] + [DOPC] = 40 and 30 mM. The curves are on an absolute scale and normalized with respect to total concentrations. The same plots with error bars are provided in the Supporting Information.

Small-angle X-ray scattering (SAXS) was employed to investigate the core–shell structure of the micelles in more detail. The normalized SAXS intensity versus scattering vector modulus of samples with rodlike micelles is plotted in Figure 3. A distinct oscillation peak in the data indicates a typical micellar core–shell structure due to the high electron density of the phosphorous atoms, leading to a high scattering contrast of the head-group shell compared to the micellar core consisting of hydrocarbon tails. The curves were fitted using the same form factor (polydisperse rods) as the corresponding SANS data, but with an additional core–shell structure of the elliptical cross section taken into account.

Due to the limited *Q*-range, it was not possible to determine the size of micelles with our SAXS equipment. However, the SAXS curves contain information on the core–shell structure of the cross-sectional dimensions, and the results from the fitting analysis are summarized in Table S1. The core of the rods roughly corresponds to the hydrocarbon tails of the phospholipid and the drug, and its dimensions were determined to be about *a* = 0.9 nm and *b* = 1.9 nm with a head-group shell equal to about 1.3 nm. The discrepancy in the total size of cross-sectional dimensions as observed with SAXS and SANS has been attributed to the additional contribution from the diffuse layer of counterions around the micelles to the SAXS profile, while mainly the core of the micelles is observed with SANS.^25,26^

### 3.2 Transition from Micelles to Bilayer Disks and Vesicles

The SANS data for the sample [*X*_PL_ = 0.25, *c*_t_ = 20 mM] were best fitted with a model for coexisting micelles, bilayer disks, and vesicles, indicating that the system has reached the point of transition from micelles to bilayer aggregates. Disklike aggregates contribute with 85% to the scattered intensity, vesicles with 15%, and micelles with 0.2%. However, since the bilayer aggregates are significantly larger than the micelles, and the contribution to intensity is proportional to the aggregate volume squared, the mass fraction of the latter type of aggregates must be considerably larger than 0.2%. In the sample [*X*_PL_ = 0.20, *c*_t_ = 20 mM], only micelles could be observed, whereas no micelles could be seen at all in [*X*_PL_ = 0.20, *c*_t_ = 10 mM], indicating a micelle-to-bilayer transition somewhere between 10 and 20 mM. The presence of a coexistence region was supported by complementary cryo-TEM measurements [c.f. Figure S7 in the Supporting information], confirming the presence of vesicles, disks, and elongated micelles in the sample [*X*_PL_ = 0.25, *c*_t_ = 20 mM].

It has previously been demonstrated that the mole fraction of the phospholipid (*x*_PL_) in mixed drug surfactant/phospholipid aggregates is generally different from the overall mole fraction in the solution (*X*_PL_), at sufficiently dilute concentrations.^27,28^ It stems from the fact that the concentrations of the free drug surfactant and free phospholipid molecules differ significantly so that the molar ratio of the phospholipid inside the aggregates is always higher than that in total (*x*_PL_ > *X*_PL_). Upon dilution, especially at concentrations below CMC of AMT, *x*_PL_ increasingly shifts to higher values, meaning drug molecules leave the aggregates.^29^

The lipid-to-drug ratio largely determines the structural properties of the aggregates. Hence, we are able to conclude that the size of micelles increases and, eventually, a transition from micelles to bilayer aggregates occurs as the fraction of phospholipids increases upon dilution of our samples. The molecules are expected to be nonuniformly distributed in the micelles, the drug molecules, with a higher spontaneous curvature, prefer to be located in the end caps of the rodlike micelles, whereas the phospholipids are enriched in the less curved central part of the rods.^30,31^

The compositions *x*_PL_ in the aggregates, as estimated from model calculations, are given in Table 1 for the different samples. The calculations are based on solution thermodynamics assuming ideal behavior for the system and have been described in detail elsewhere.^28,29^ The point where bilayer aggregates start to form in the *X*_PL_ = 0.25 series may be estimated to occur between *x*_PL_ = 0.39 (*c*_t_ = 30 mM, only micelles present) and *x*_PL_ = 0.47 (*c*_t_ = 20 mM, micelles + bilayers). Likewise, from the behavior in the *X*_PL_ = 0.20 series, we may conclude that the transition must occur in the interval 0.42–0.62. Since the point of transition *x*_PL_^*^ is expected to be about the same for the two dilution series, we may conclude that it is located in the interval *x*_PL_^*^ = 0.42–0.47.

**Table 1:** Calculated Mole Fractions of DOPC Inside the Aggregates, *x*_PL_, for Samples with Different Total Concentrations at Two Dilution Series

*X*_PL_ = 0.20			*X*_PL_ = 0.25		
*c*_t_ (mM)	*x*_PL_	aggregate	*c*_t_ (mM)	*x*_PL_	aggregate
40	0.29	micelles	40	0.35	micelles
30	0.33	micelles	30	0.39	micelles
20	0.42	micelles	20	0.47*	micelles + vesicles + disks
10	0.62*	vesicles + disks	10	0.65	vesicles + disks
5	0.79	vesicles	5	0.81	vesicles

We have recently demonstrated that conventional surfactants with a flexible aliphatic hydrocarbon chain as a tail usually have a transition point well below *x*_PL_^*^ = 0.5, whereas bile salt surfactants have a transition point *x*_PL_^*^ > 0.5.^29^ Our present result for the drug surfactant AMT indicates an intermediate capacity to dissolve phospholipids in mixed micelles. Drug surfactants such as AMT resemble bile salt surfactants, in that they consist of a rigid hydrophobic tail but lack additional hydrophilic hydroxyl groups that are common in bile salts.

### 3.3 Vesicular Region

The SANS results for samples containing bilayer aggregates are summarized in Table 2. In the coexistence region, the sizes of vesicles and disks are comparatively large, as is the fraction of disks. As a matter of fact, the dimensions of the disks are too large to be determined from our SANS data. As the samples are diluted, the vesicles significantly decrease in size, as does the fraction of disks. Most interestingly and somewhat counterintuitively, the vesicles decrease in size with an increasing fraction of the phospholipid in the aggregates, i.e., the opposite trend to what we observe for micelles. In the samples with a concentration of 10 mM in both series, unilamellar vesicles are the predominant structure and they have shrunk to sizes with an average diameter of smaller than 30 nm.

**Table 2:** Results of SANS and SAXS Data Analysis for Mixtures of AMT and DOPC below the Point of Micelle-to-bilayer Transitions

	*X*_PL_ = 0.25	*X*_PL_ = 0.20	*X*_PL_ = 0.25	*X*_PL_ = 0.20	*X*_PL_ = 0.25
parameter	*c_t_* = 20 mM	*c_t_* = 10 mM	*c_t_* = 10 mM	*c_t_* = 5 mM	*c_t_* = 5 mM
disk radius (nm)		28.5 ± 0.4	30.6 ± 0.4		
bilayer half-thickness (nm)	1.5 ± 0.1	1.8 ± 0.1	1.8 ± 0.1		
vesicle radius (nm)	31.5 ± 0.1	13.0 ± 0.1	11.3 ± 0.1	7.6 ± 0.1	8.1 ± 0.1
fraction^a^ of vesicles	0.15 ± 0.01	0.75 ± 0.01	0.75 ± 0.02	1	1
polydispersity of vesicles	0.42 ± 0.01	0.33 ± 0.01	0.39 ± 0.01	0.38 ± 0.01	0.38 ± 0.01
fraction^a^ of micelles	0.002 ± 0.001				

a Intensity-weighted fraction.

**Figure 4:**
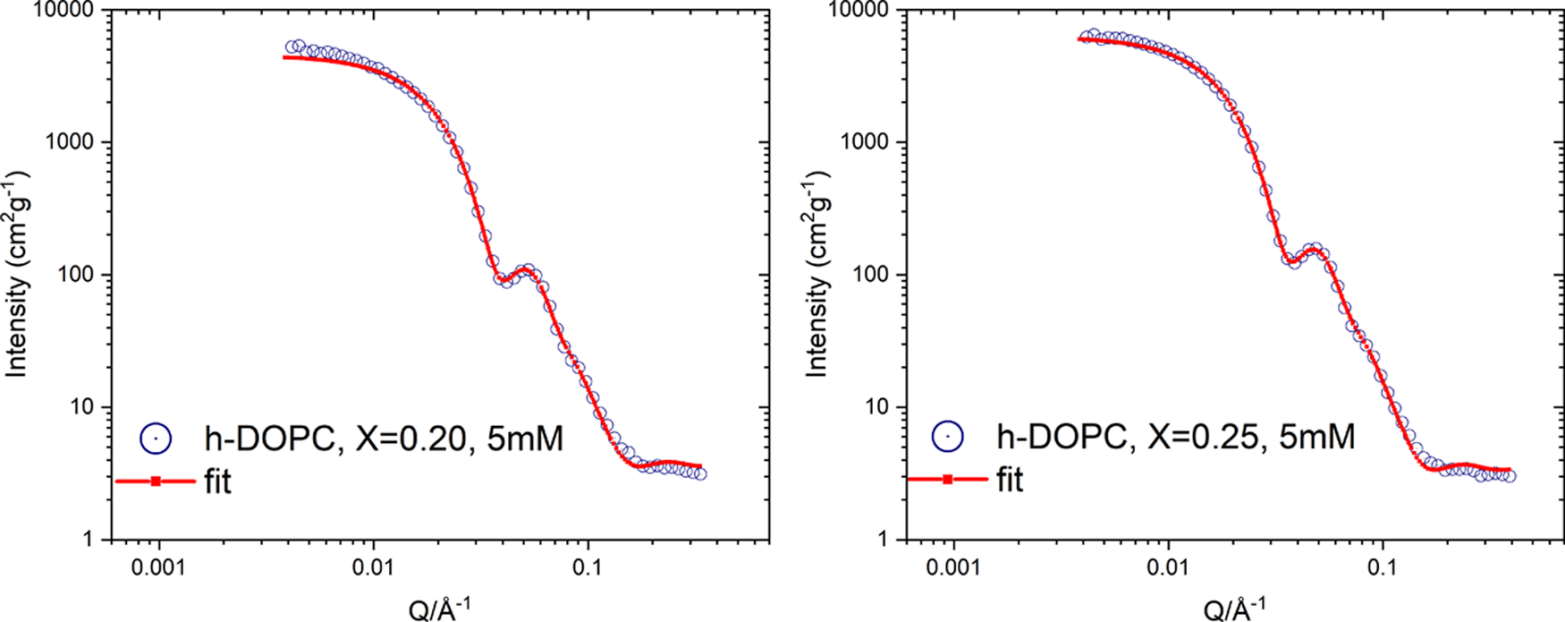
SANS profiles for the samples with DOPC mole ratios of *X* = 0.20 and 0.25 and a total concentration ([AMT] + [DOPC]) of 5 mM. The curves are on an absolute scale and normalized against the total concentrations.

**Figure 5:**
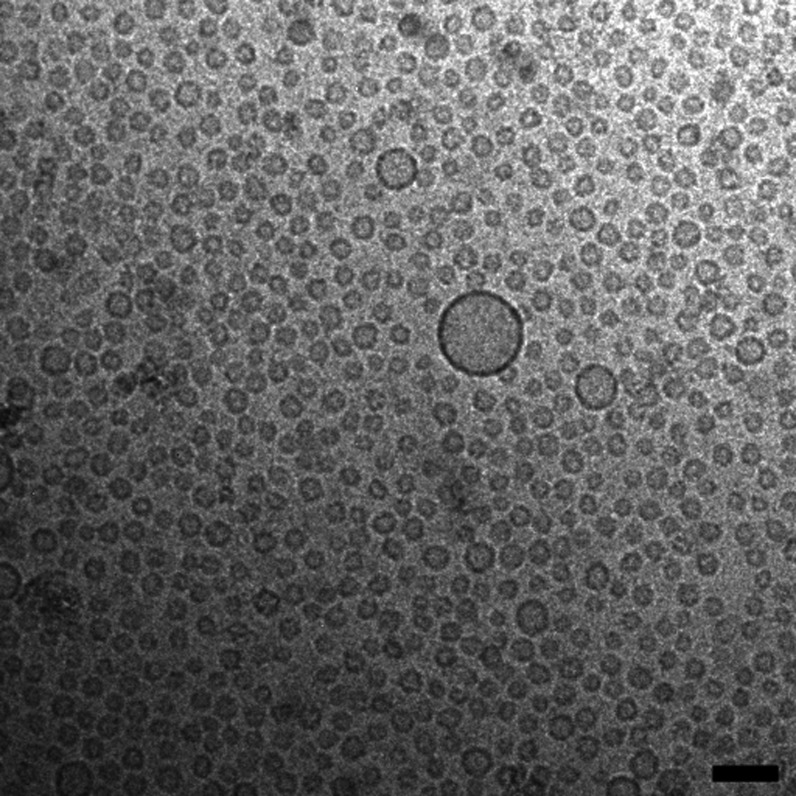
Cryo-TEM image of the AMT-DOPC sample [*X*_PL_ = 0.25, *c*_t_ = 5 mM] showing small unilamellar vesicles with an average diameter of about 15 nm. The scale bar is 50 nm.

When further diluting our samples to 5 mM, only small vesicles are present without any disks. SANS data fitted with a model for polydisperse unilamellar vesicles are shown in Figure 4. The average diameter of the vesicles is smaller than 20 nm for both samples. These vesicles are smaller than the ones usually reported, whether naturally occurring liposomes or synthetically prepared or spontaneously formed unilamellar vesicles, and we have chosen to denote them ultrasmall unilamellar vesicles. The conspicuously small vesicles are shown in a cryo-TEM image in Figure 5 for the sample [*X*_PL_ = 0.25, *c*_t_ = 5 mM], where the size of the vesicles as determined with SANS is confirmed. The unilamellar structure of the vesicles is clearly seen and the majority of vesicles have a size close to 15 nm in diameter, although a few larger vesicles are also observed. The polydispersity of the vesicles expressed in terms of relative standard deviation was determined from our SANS analysis to be in the interval σ_*R*_/⟨*R*⟩ = 0.35–0.45. These numbers agree very well with what has previously been reported for spontaneously formed vesicles in mixed surfactant systems^13,32^ as well as for phospholipid liposomes.^33,34^

Unlike sonicated/extruded phospholipid liposomes that are metastable and fuse to larger aggregates, eventually sedimenting and forming stacks of large bilayer structures,^35^ the observed ultrasmall vesicles are stable for several months and appear to be the equilibrium structure at low solute concentrations and certain phospholipid–drug compositions.

#### 3.3.1 Comparison between AMT-DOPC and AMT-DMPC Vesicles

To investigate the impact of the structure of the phospholipid tail on the size of unilamellar vesicles, we have compared cryo-TEM images of the sample [*X*_PL_ = 0.25, *c*_t_ = 5 mM] at 37 °C in D_2_O with AMT and either DOPC (18:1, i.e., two tails with 18 carbons and 1 double bond) or DMPC (14:0) as a phospholipid. Since the critical aggregate concentration of the phospholipid is always several orders of magnitude lower than that for AMT, the concentrations of vesicles and free AMT are expected to be more or less identical in the two samples. The temperature was chosen to be 37 °C, in order to be well above the lipid phase-transition temperature of DMPC. The size of ultrasmall AMT-DOPC vesicles was found to be about the same as observed at 21 °C. Most interestingly, the vesicles formed in the AMT-DMPC system were considerably larger in size than AMT-DOPC vesicles, i.e., about 15 nm in diameter compared to about 60 nm in the corresponding AMT-DMPC sample as estimated from our cryo-TEM images (cf. Figure S8 in the Supporting Information).

#### 3.3.2 Comparison with Vesicles Formed by Oppositely Charged Surfactants

Small unilamellar vesicles formed spontaneously by two oppositely charged surfactants are generally believed to be equilibrium structures. It is interesting to compare these kinds of systems with our present system of vesicles formed in mixtures of a drug surfactant (or a surfactant with a rigid tail) and a phospholipid. In both systems, micelles or small unilamellar vesicles, depending on composition, are formed spontaneously by simply mixing the components. In particular, rather small vesicles are formed spontaneously through the dilution of samples in a micellar regime and the vesicles are stable for several months (or as long as the components are chemically stable). The size of vesicles in both systems largely depends on the detailed chemical structure of the components, and in both systems, ultrasmall unilamellar vesicles, with diameters of less than about 30 nm, have been found in mixtures with certain structural properties of the components. For instance, conspicuously small unilamellar vesicles have been observed in mixtures of a single-tailed anionic surfactant and a double-tailed cationic surfactant^13^ and in mixtures of a cationic surfactant with a long hydrocarbon tail and an anionic surfactant with a short fluorocarbon tail.^36^

There are also certain differences between the two types of systems. In anionic–cationic surfactant mixtures, rather small unilamellar vesicles can be found in pure water or at low ionic strengths. However, addition of a considerable amount of salt tends to make the vesicles larger in size, as well as increasing the fraction of geometrically open disks.^37^ In contrast, small and ultrasmall unilamellar vesicles may be formed spontaneously at very high electrolyte concentrations in the drug surfactant–phospholipid system. Moreover, in the anionic–cationic surfactant system, the size of vesicles and disks tend to increase with decreasing surfactant concentration, as does the fraction of disks in the samples. On the contrary, in our present drug surfactant–phospholipid system, the vesicles tend to decrease in size with decreasing surfactant–phospholipid concentration.

In both systems, the formation of small unilamellar vesicles is facilitated by mixing two amphiphilic components that display a certain asymmetry. In the anionic–cationic surfactant system, there is an asymmetry with respect to the head-group charge number. As a result, surfactant molecules will distribute between the outer and inner monolayers of the vesicles, in order to give the positively charged outer layer a higher surface charge density. This will reduce the bending rigidity of the bilayer and enable the formation of comparatively small vesicles.^38,39^

We have recently demonstrated that surfactants with a rigid tail may possess an exceptionally high spontaneous curvature, whereas phospholipids have a low, sometimes even negative, spontaneous curvature.^29^ Hence, the large asymmetry with respect to spontaneous curvature makes the drug molecules prefer the outer vesicle leaflet, whereas the phospholipid prefers to be located in the inner leaflet. As a result, the bilayer bending rigidity will be substantially reduced,^31,40,41^ enabling the formation of ultrasmall unilamellar vesicles. The fact that the spontaneous curvature is expected to decrease with increasing volume of the lipophilic part of phospholipids may explain why DOPC to a higher extent than DMPC prefers to be located in the inner vesicle leaflets and that vesicles formed in the AMT-DOPC system are considerably smaller than those in the AMT-DMPC system. The reduced flexibility of the unsaturated tails of DOPC may also contribute to the bending properties and size of unilamellar vesicles. In addition to bending properties, the size of aggregates formed by self-assembling amphiphilic molecules is influenced by the thermodynamics of self-assembly. Hence, equilibrium vesicles are expected to grow in size with increasing amphiphile solute concentration in a similar manner to the growth behavior of surfactant micelles. This effect could, at least partly, explain the conspicuous reduction in vesicle size upon diluting our samples.

## 4 Conclusions

We have investigated the structural properties of self-assembled aggregates in mixtures of the drug surfactant AMT and the phospholipid DOPC in physiological salt concentrations using the complementary techniques SANS, SAXS, and cryo-TEM. Rodlike micelles with an elliptical cross section are formed in samples with a total concentration of the drug and the phospholipid equal to 40 mM at drug-to-phospholipid molar ratios 3:1 and 4:1. The micelles grow in length when the samples are diluted, and below about the critical micelle concentration of AMT (CMC ≈ 17 mM), the micelles are transformed into unilamellar bilayer vesicles and disks. In one of our samples, SANS data analysis demonstrates the presence of coexisting micelles, vesicles, and disks, and the observation is confirmed with cryo-TEM.

When further diluting the samples to 10 and 5 mM, the vesicles become significantly smaller, while the fraction of bilayer disks decreases. In the two samples at 5 mM, only unilamellar vesicles with an average diameter of about 15 nm are present. Most interestingly, these ultrasmall vesicles are formed spontaneously by simple dilution of micellar solutions. To our knowledge, such small vesicles have previously never been reported to be formed spontaneously in a phospholipid-based system. The ultrasmall vesicles are stable and maintain their conspicuously small size for at least several months.

The AMT-DOPC system much resembles mixed anionic–cationic surfactant systems. In both types of systems, small unilamellar vesicles are formed spontaneously by simply diluting samples in the micellar regime. The size of the vesicles in both types of systems is a strong function of surfactant composition and concentration as well as the chemical structure of amphiphilic molecules. A conspicuous difference between the two types of systems is that vesicles formed by AMT and DOPC decrease in size with decreasing total amphiphile concentration. As a matter of fact, the mixed AMT-DOPC vesicles tend to become even smaller than the smallest ones reported for any anionic–cationic system. Their size is also significantly smaller than that reported for naturally occurring extracellular vesicles and we have chosen to denote them ultrasmall unilamellar vesicles, defined as unilamellar vesicles with diameters of lower than 20 nm. Ultrasmall vesicles composed of biocompatible components in physiological saline solution combine good solubilization capacity with excellent transport properties, making this kind of system a promising candidate for applications in the fields of biotechnology and drug delivery.

## Supporting Information Available

SANS results of fitted *X*_PL_ = 0.20 and 0.25 samples; SAXS data analysis results (Table S1); SANS data for the sample [*X*_PL_ = 0.25, *c*_t_ = 5 mM] fitted with different models; SAXS data with added error bars; cryo-TEM image of the samples [*X*_PL_ = 0.25, *c*_t_ = 5 mM] and [*X*_PL_ = 0.25, *c*_t_ = 20 mM]; cryo-TEM images of vesicles formed in AMT-DOPC and AMT-DMPC; description of employed models for fitting SANS and SAXS data; description of the thermodynamic model used to calculate aggregate mole fractions; and related references (PDF)

## References

[ref1] Rideau E.; Dimova R.; Schwille P.; et al. Liposomes and polymersomes: a comparative review towards cell mimicking. Chem. Soc. Rev. 2018, 47, 8572–8610. 10.1039/C8CS00162F. 30177983

[ref2] Doyle L. M.; Wang M. Z. Overview of extracellular vesicles, their origin, composition, purpose, and methods for exosome isolation and analysis. Cells 2019, 8, 727 10.3390/cells8070727. 31311206 PMC6678302

[ref3] Li J.; Wang X.; Zhang T.; et al. A review on phospholipids and their main applications in drug delivery systems. Asian J. Pharm. Sci. 2015, 10, 81–98. 10.1016/j.ajps.2014.09.004.

[ref4] Vader P.; Mol E. A.; Pasterkamp G.; et al. Extracellular vesicles for drug delivery. Adv. Drug Delivery Rev. 2016, 106, 148–156. 10.1016/j.addr.2016.02.006. 26928656

[ref5] Tenchov R.; Bird R.; Curtze A. E.; et al. Lipid Nanoparticles— From Liposomes to mRNA Vaccine Delivery, a Landscape of Research Diversity and Advancement. ACS Nano 2021, 15, 16982–17015. 10.1021/acsnano.1c04996. 34181394

[ref6] Jesorka A.; Orwar O. Liposomes: technologies and analytical applications. Annu. Rev. Anal. Chem. 2008, 1, 801–832. 10.1146/annurev.anchem.1.031207.112747. 20636098

[ref7] Šegota S.; Težak D. Spontaneous formation of vesicles. Adv. Colloid Interface Sci. 2006, 121, 51–75. 10.1016/j.cis.2006.01.002. 16769012

[ref8] Pedersen J. S.; Egelhaaf S. U.; Schurtenberger P. Formation of polymerlike mixed micelles and vesicles in lecithin-bile salt solutions: a small-angle neutron-scattering study. J. Phys. Chem. A 1995, 99, 1299–1305. 10.1021/j100004a033.

[ref9] Almgren M. Mixed micelles and other structures in the solubilization of bilayer lipid membranes by surfactants. Biochim. Biophys. Acta, Biomembr. 2000, 1508, 146–163. 10.1016/S0005-2736(00)00309-6. 11090823

[ref10] Viseu M. I.; Correia R. F.; Fernandes A. C. Time evolution of the thermotropic behavior of spontaneous liposomes and disks of the DMPC–DTAC aqueous system. J. Colloid Interface Sci. 2010, 351, 156–165. 10.1016/j.jcis.2010.06.061. 20701924

[ref11] Kaler E. W.; Herrington K. L.; Murthy A. K.; et al. Phase behavior and structures of mixtures of anionic and cationic surfactants. J. Phys. Chem. A 1992, 96, 6698–6707. 10.1021/j100195a033.

[ref12] Tondre C.; Caillet C. Properties of the amphiphilic films in mixed cationic/anionic vesicles: a comprehensive view from a literature analysis. Adv. Colloid Interface Sci. 2001, 93, 115–134. 10.1016/S0001-8686(00)00081-6. 11591107

[ref13] Bergström M.; Pedersen J. S. A Small-angle neutron scattering study of surfactant aggregates formed in aqueous mixtures of sodium dodecyl sulfate and didodecyldimethylammonium bromide. J. Phys. Chem. B 2000, 104, 4155–4163. 10.1021/jp993666s.

[ref14] Jung H.-T.; Lee S. Y.; Kaler E. W.; et al. Gaussian curvature and the equilibrium among bilayer cylinders, spheres, and discs. Proc. Natl. Acad. Sci. U.S.A. 2002, 99, 15318–15322. 10.1073/pnas.242374499. 12444257 PMC137714

[ref15] Efthymiou C.; Bergström L. M.; Pedersen J. N.; et al. Self-assembling properties of ionisable amphiphilic drugs in aqueous solution. J. Colloid Interface Sci. 2021, 600, 701–710. 10.1016/j.jcis.2021.05.049. 34049025

[ref16] Motlaq V. F.; Adlmann F.; Hernández V. A.; et al. Dissolution mechanism of supported phospholipid bilayer in the presence of amphiphilic drug investigated by neutron reflectometry and quartz crystal microbalance with dissipation monitoring. Biochim. Biophys. Acta, Biomembr. 2022, 1864, 183976 10.1016/j.bbamem.2022.183976. 35662645

[ref17] Venkatesan G. A.; Taylor G. J.; Basham C. M.; et al. Evaporation-induced monolayer compression improves droplet interface bilayer formation using unsaturated lipids. Biomicrofluidics 2018, 12, 024101 10.1063/1.5016523. 29576833 PMC5832467

[ref18] Thureau A.; Roblin P.; Perez J. BioSAXS on the SWING beamline at Synchrotron SOLEIL. J. Appl. Crystallogr. 2021, 54, 1698–1710. 10.1107/S1600576721008736.

[ref19] ZOOM Diffractometer at the ISSI Neutron Source. https://www.isis.stfc.ac.uk/Pages/Zoom.aspx.

[ref20] Lindner P.; Zemb T. Neutron, X-ray and Light Scattering: Introduction to an Investigative Tool for Colloidal and Polymeric Systems; North-Holland: Netherlands, 1991.

[ref21] http://www.mantidproject.org/Main_Page.

[ref22] Pedersen J. S. Analysis of small-angle scattering data from colloids and polymer solutions: modeling and least-squares fitting. Adv. Colloid Interface Sci. 1997, 70, 171–210. 10.1016/S0001-8686(97)00312-6.

[ref23] Almgren M.; Edwards K.; Karlsson G. Cryo transmission electron microscopy of liposomes and related structures. Colloids Surf., A 2000, 174, 3–21. 10.1016/S0927-7757(00)00516-1.

[ref24] Kučerka N.; Tristram-Nagle S.; Nagle J. F. Structure of fully hydrated fluid phase lipid bilayers with monounsaturated chains. J. Membr. Biol. 2006, 208, 193–202. 10.1007/s00232-005-7006-8. 16604469

[ref25] Aswal V. K.; Goyal P. S.; Amenitsch H.; et al. Counterion condensation in ionic micelles as studied by a combined use of SANS and SAXS. Pramana 2004, 63, 333–338. 10.1007/BF02704994.

[ref26] Iampietro D. J.; Brasher L. L.; Kaler E. W.; et al. Direct analysis of SANS and SAXS measurements of catanionic surfactant mixtures by fourier transformation. J. Phys. Chem. B 1998, 102, 3105–3113. 10.1021/jp973326b.

[ref27] Lichtenberg D. Characterization of the solubilization of lipid bilayers by surfactants. Biochim. Biophys. Acta, Biomembr. 1985, 821, 470–478. 10.1016/0005-2736(85)90052-5. 4074739

[ref28] Bergström L. M.; Aratono M. Synergistic effects in mixtures of two identically charged ionic surfactants with different critical micelle concentrations. Soft Matter 2011, 7, 8870–8879. 10.1039/c1sm06064c.

[ref29] Forooqi Motlaq V.; Ortega-Holmberg M.; Edwards K.; et al. Investigation of the enhanced ability of bile salt surfactants to solubilize phospholipid bilayers and form mixed micelles. Soft Matter 2021, 17, 7769–7780. 10.1039/D1SM00745A. 34351343

[ref30] Madenci D.; Salonen A.; Schurtenberger P.; et al. Simple model for the growth behaviour of mixed lecithin–bile salt micelles. Phys. Chem. Chem. Phys. 2011, 13, 3171–3178. 10.1039/C0CP01700K. 21135948

[ref31] Bergström L. M. Bending elasticity of charged surfactant layers: The effect of mixing. Langmuir 2006, 22, 6796–6813. 10.1021/la060520t. 16863224

[ref32] Bergström M.; Pedersen J. S.; Schurtenberger P.; et al. Small-angle neutron scattering (SANS) study of vesicles and lamellar sheets formed from mixtures of an anionic and a cationic surfactant. J. Phys. Chem. B 1999, 103, 9888–9897. 10.1021/jp991846w.

[ref33] Hallett F. R.; Nickel B.; Samuels C.; et al. Determination of vesicle size distributions by freeze-fracture electron microscopy. J. Electron Microsc. Tech. 1991, 17, 459–466. 10.1002/jemt.1060170409. 1865244

[ref34] Kiselev M.; Zemlyanaya E.; Aswal V. SANS study of the unilamellar DMPC vesicles The fluctuation model of lipid bilayer 2004. arXiv preprint physics/0411047.

[ref35] Winterhalter M.; Lasic D. D. Liposome stability and formation: experimental parameters and theories on the size distribution. Chem. Phys. Lipids 1993, 64, 35–43. 10.1016/0009-3084(93)90056-9. 8242841

[ref36] Jung H.; et al. Gaussian curvature and the equilibrium between cylinders, discs and spheres. Proc. Natl. Acad. Sci. U.S.A. 2002, 99, 15318–15322. 10.1073/pnas.242374499. 12444257 PMC137714

[ref37] Bergström L. M.; Skoglund S.; Edwards K.; et al. Spontaneous transformations between surfactant bilayers of different topologies observed in mixtures of sodium octyl sulfate and hexadecyltrimethylammonium bromide. Langmuir 2014, 30, 3928–3938. 10.1021/la4042259. 24697326

[ref38] Bergström M. Thermodynamics of vesicle formation from a mixture of anionic and cationic surfactants. Langmuir 1996, 12, 2454–2463. 10.1021/la950873k.

[ref39] Bergström L. M. Bending elasticity of charged surfactant layers: The effect of mixing. Langmuir 2006, 22, 6796–6813. 10.1021/la060520t. 16863224

[ref40] Safran S. A.; Pincus P.; Andelman D. Theory of spontaneous vesicle formation in surfactant mixtures. Science 1990, 248, 354–356. 10.1126/science.248.4953.354. 17784490

[ref41] Kozlov M. M.; Helfrich W. Effects of a cosurfactant on the stretching and bending elasticities of a surfactant monolayer. Langmuir 1992, 8, 2792–2797. 10.1021/la00047a035.

