# Supplementary material for: Spontaneous Formation of Ultrasmall Unilamellar Vesicles in Mixtures of an Amphiphilic Drug and a Phospholipid
Source: Langmuir. 2023 Aug 2;39(32):11337–44. doi: 10.1021/acs.langmuir.3c01023 (PMC10433524; doi:10.1021/acs.langmuir.3c01023)
Supplement: Supplementary file 1 — la3c01023_si_001.pdf [file la3c01023_si_001.pdf]

Spontaneous formation of ultra-small unilamellar vesicles in mixtures of an amphiphilic drug and a phospholipid

Vahid Forooqi Motlaq[*¤], Lars Gedda[¥], Katarina Edwards[¥], James Doutch[§], and L. M. Bergström[*¤]

*) Department of medicinal chemistry, Uppsala University, P.O. Box 547, 751 23, Uppsala, Sweden
¤) Department of Pharmacy, Uppsala University, P.O. Box 580, 75123 Uppsala, Sweden
¥) Department of Chemistry-Ångström, P.O. Box 573,, Uppsala University, 751 23 Uppsala, Sweden
§) ISIS Neutron and Muon Source, STFC, Rutherford Appleton Laboratory, Harwell Campus, Didcot, Oxon, UK

1. Small angle neutron scattering data

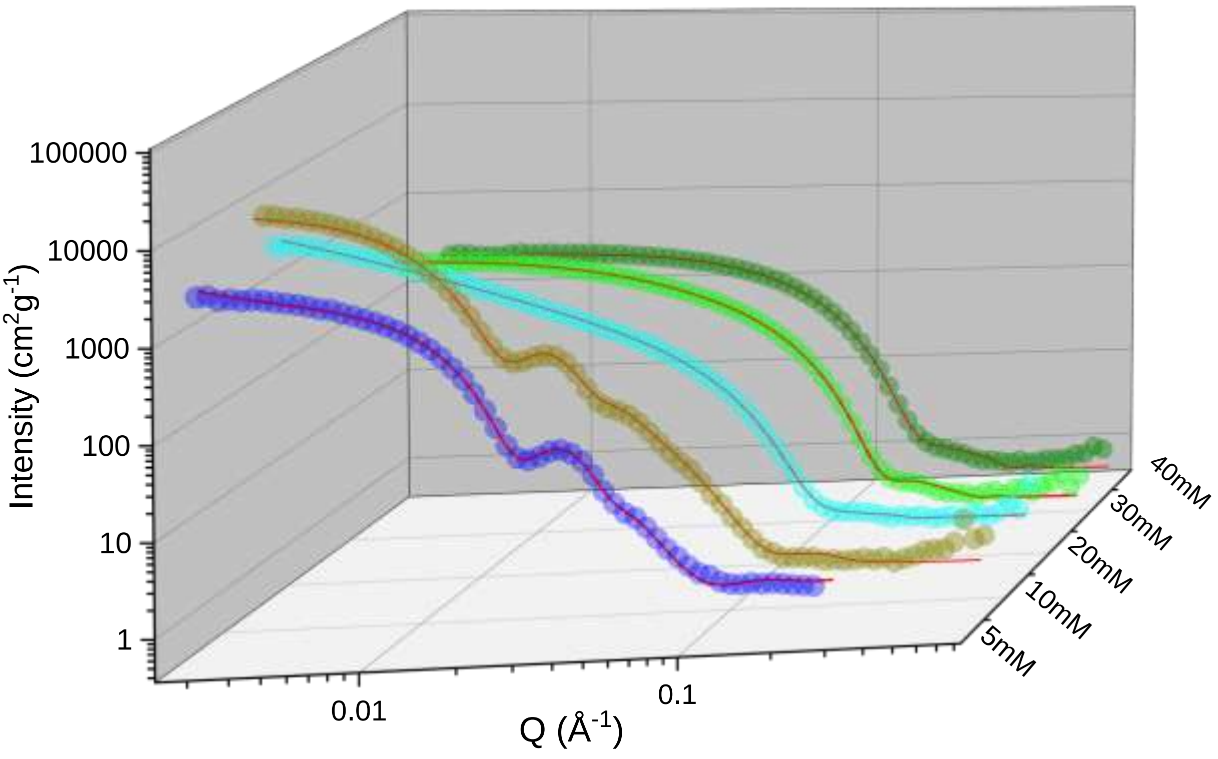

Figure S1: 3D demonstration of SANS profiles for the samples with DOPC mole ratio of X=0.20, and total concentrations ([AMT] + [DOPC]) of 40, 30, 20 10, and 5mM. The curves are in absolute scale and normalized with respect to total concentrations.

1.2 Small angle neutron scattering data of X=0.20 series

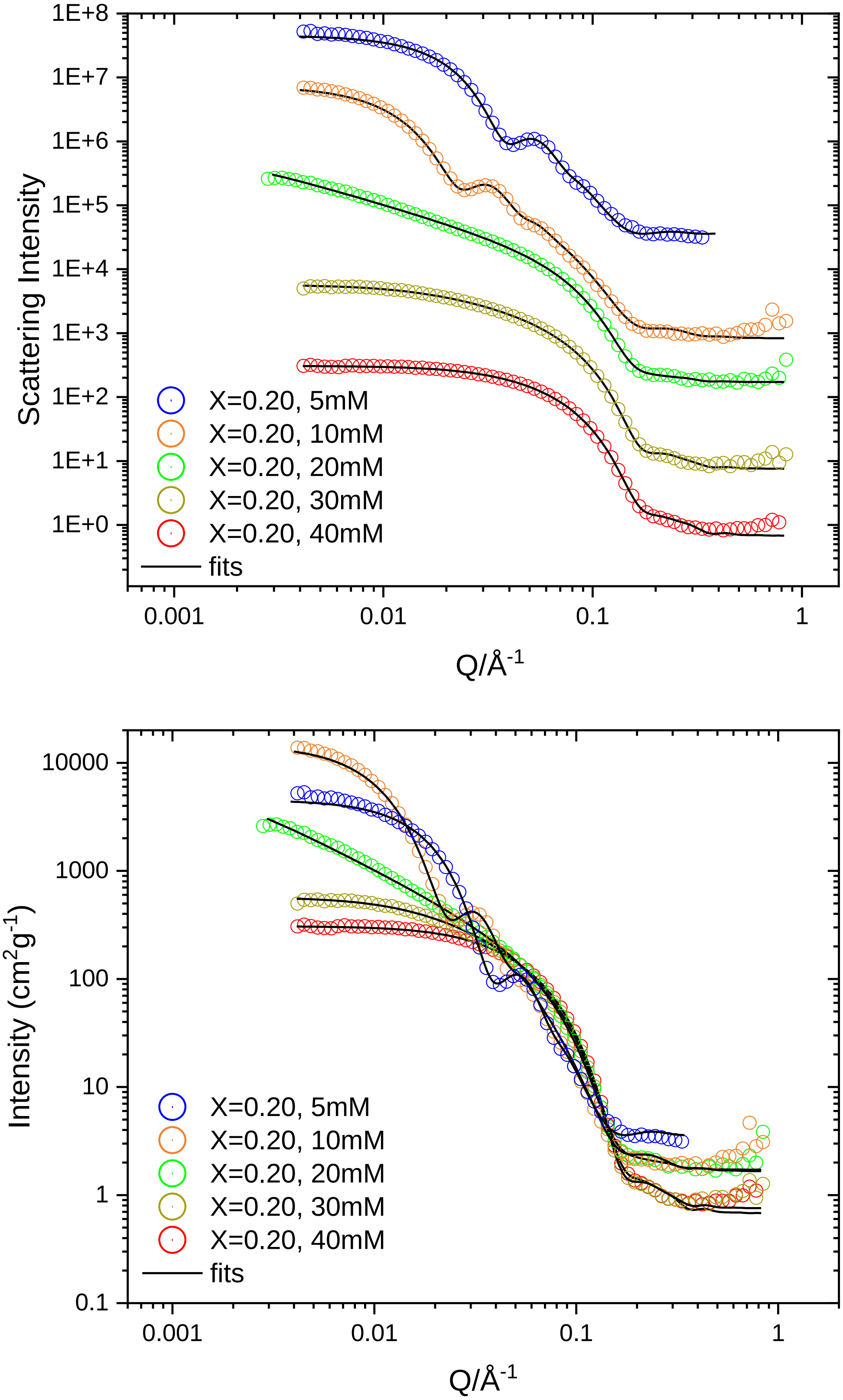

Figure S2: Top) SANS profiles for the samples with DOPC mole ratio of X=0.20, and total concentrations ([AMT]+[DOPC]) of 40, 30, 20 10, and 5mM. The curves have been set in absolute scale and normalized against the total concentrations. Bottom) the same curves that have been but separated to emphasize the quality of the fits.

1.3 Small angle neutron scattering data of X=0.25 series

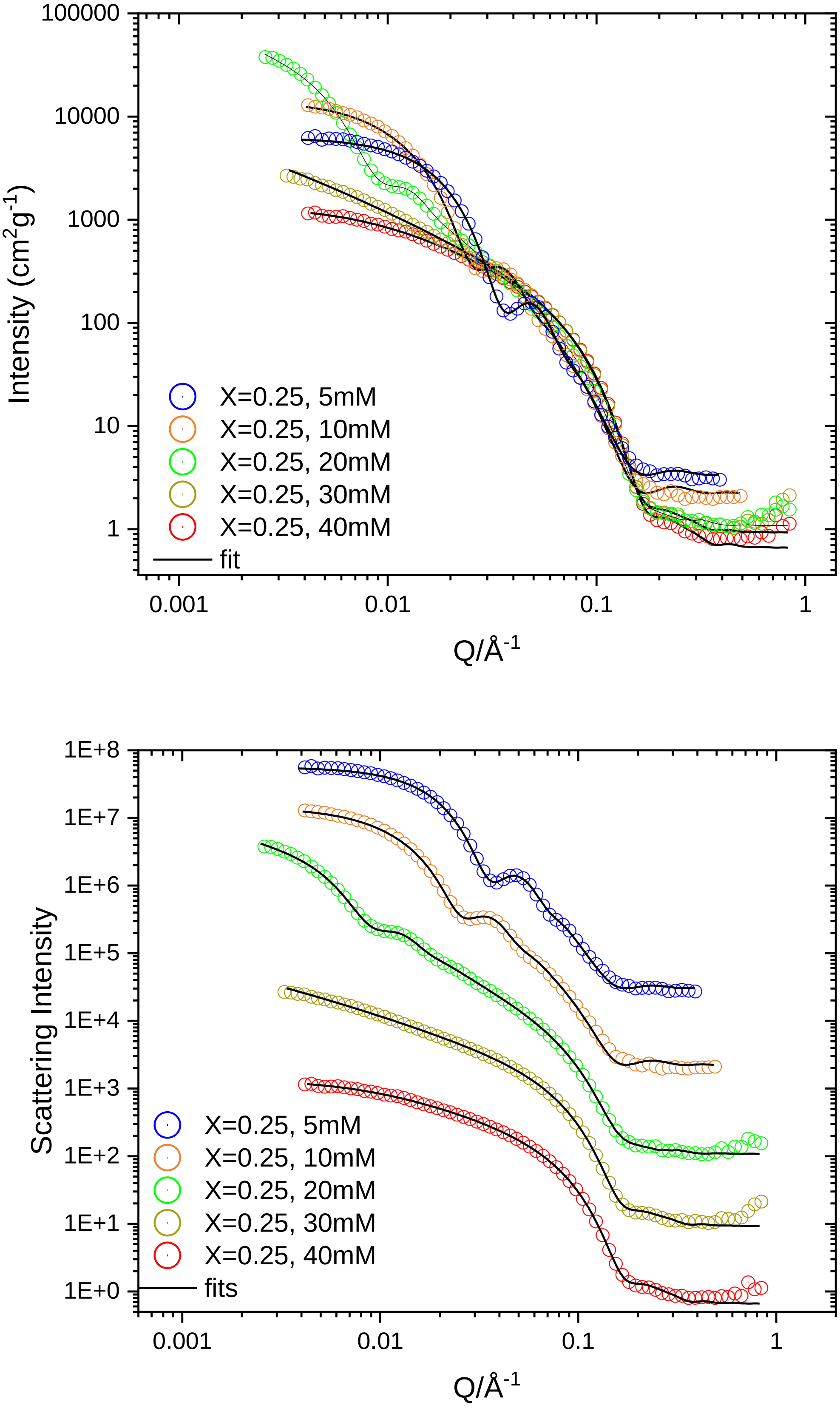

Figure S3: SANS profiles for the samples with DOPC mole ratio of X=0.25, and total concentrations ([AMT]+[DOPC]) of 40, 30, 20 10, and 5mM. The curves have been set to absolute scale and normalized against the total concentrations. Bottom) the same curves that have been but separated to emphasize the quality of the fits.

1.4 Small angle neutron scattering data of micellar region

Table S1: Results of SANS and SAXS data analyses of samples where AMT and DOPC formed micellar aggregates.

Composition	40 mM	30 mM	20 mM
X_{PL} = 0.20	$\langle L \rangle$ = 5.9 nm<br>a = 1.8 nm<br>b =2.8 nm<br>$\delta_L/\langle L \rangle$ = 0.7<br><br>a_{hc} = 0.7 nm<br>b_{hc} = 1.9 nm<br>d = 1.4 nm	$\langle L \rangle$ = 15 nm<br>a = 1.8 nm<br>b =2.7 nm<br>$\delta_L/\langle L \rangle$ = 0.7<br><br>a_{hc} = 0.8 nm<br>b_{hc} = 1.9 nm<br>d = 1.3 nm	a = 1.8 nm<br>b =2.9 nm
X_{PL} = 0.25	$\langle L \rangle$ = 31 nm<br>a = 1.8 nm<br>b =2.7 nm<br>$\delta_L/\langle L \rangle$ = 0.7<br><br>a_{hc} = 0.7 nm<br>b_{hc} = 1.7 nm<br>d = 1.3 nm	$\langle L \rangle$ = 154 nm<br>a = 1.9 nm<br>b =2.8 nm<br>$\delta_L/\langle L \rangle$ = 0.8<br><br>a_{hc} = 0.8 nm<br>b_{hc} = 1.9 nm<br>d = 1.3 nm	Micelles + Vesicles + Disks

The SANS data were fitted with a model for polydisperse rodlike micelles with weight-average length $\langle L \rangle$, relative standard deviation $\delta_L/\langle L \rangle$ and elliptical cross section with half-axes a and b. In the sample [X_{PL}= 0.20, c_t = 20mM] the micelles were too large for their size distribution to be determined from the SANS data. The SAXS data were fitted with the same model with addition of core-shell layer to the cross-section. a_{hc} and b_{hc} are the half-axes of the hydrocarbon core of the micelles and d is the thickness of the hydrophilic head-group shell.

1.5 Small angle neutron scattering data of X=0.25, 5mM sample

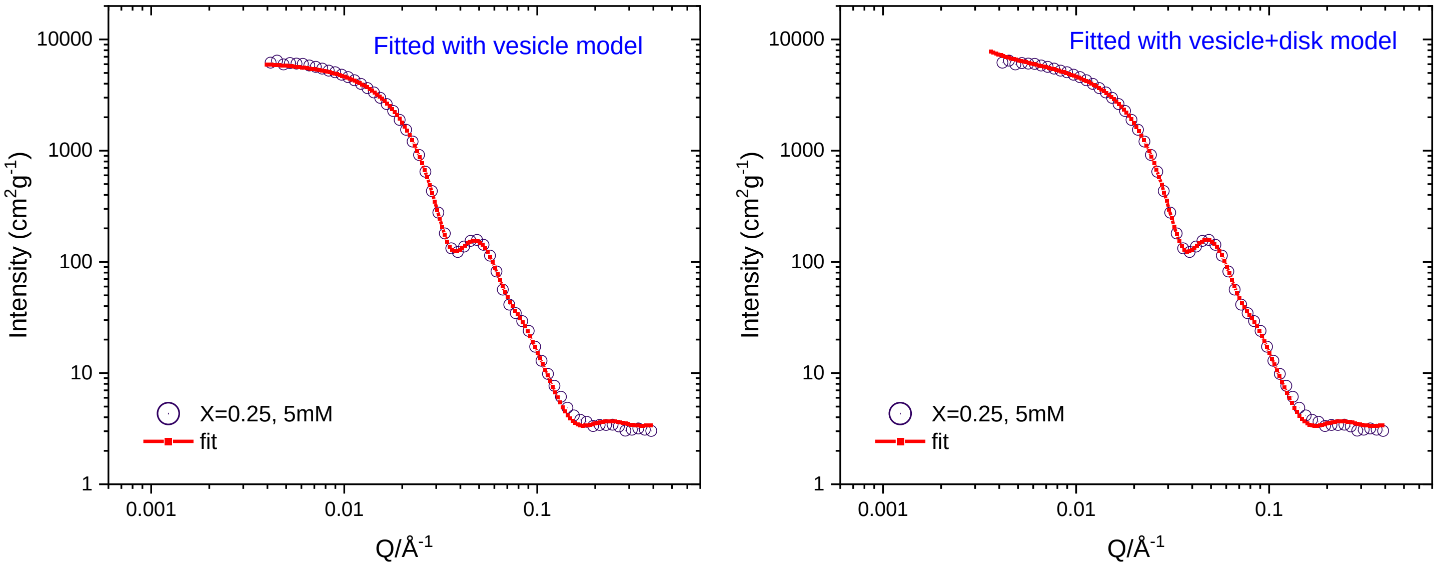

Figure S4: SANS profiles for the samples with DOPC mole ratio of X=0.25, and total concentrations ([AMT]+[DOPC]) of 5mM. The curves have been set to absolute scale and normalized against the total concentrations. left) The curve fitted with the model containing form factor of polydisperse vesicle. Right) The same curves that have been fitted with model containing form factor of polydisperse vesicle and polydisperse disk. The introduction of the presence of disks in the model does not significantly improve the quality of the fit.

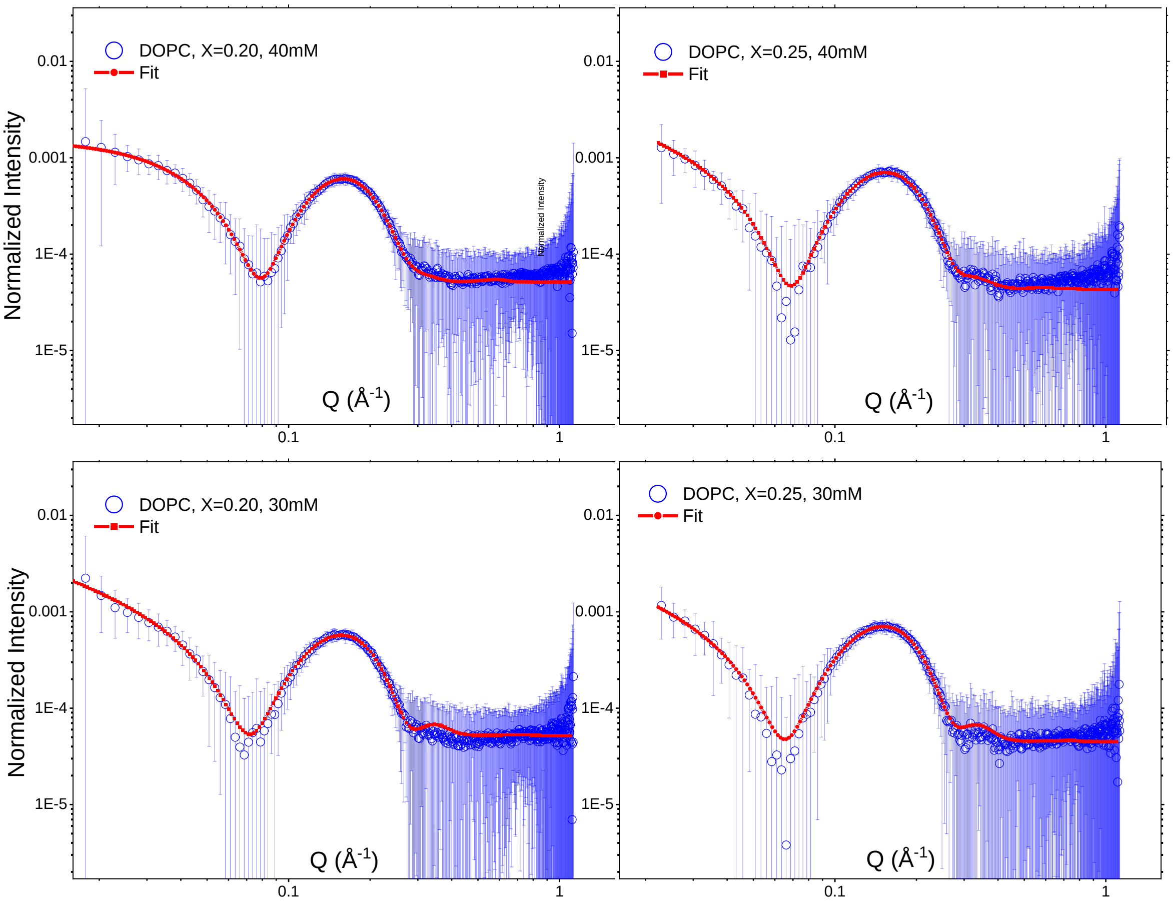

Figure S5: SAXS profiles for the samples with DOPC mole ratio of X=0.20 and 0.25, and total concentrations ([AMT]+[DOPC]) of 40, and 30 mM with added error bars. The curves are in absolute scale and normalized for the total concentrations.

2. Cryo-TEM images

2.1 X=0.25, 5mM

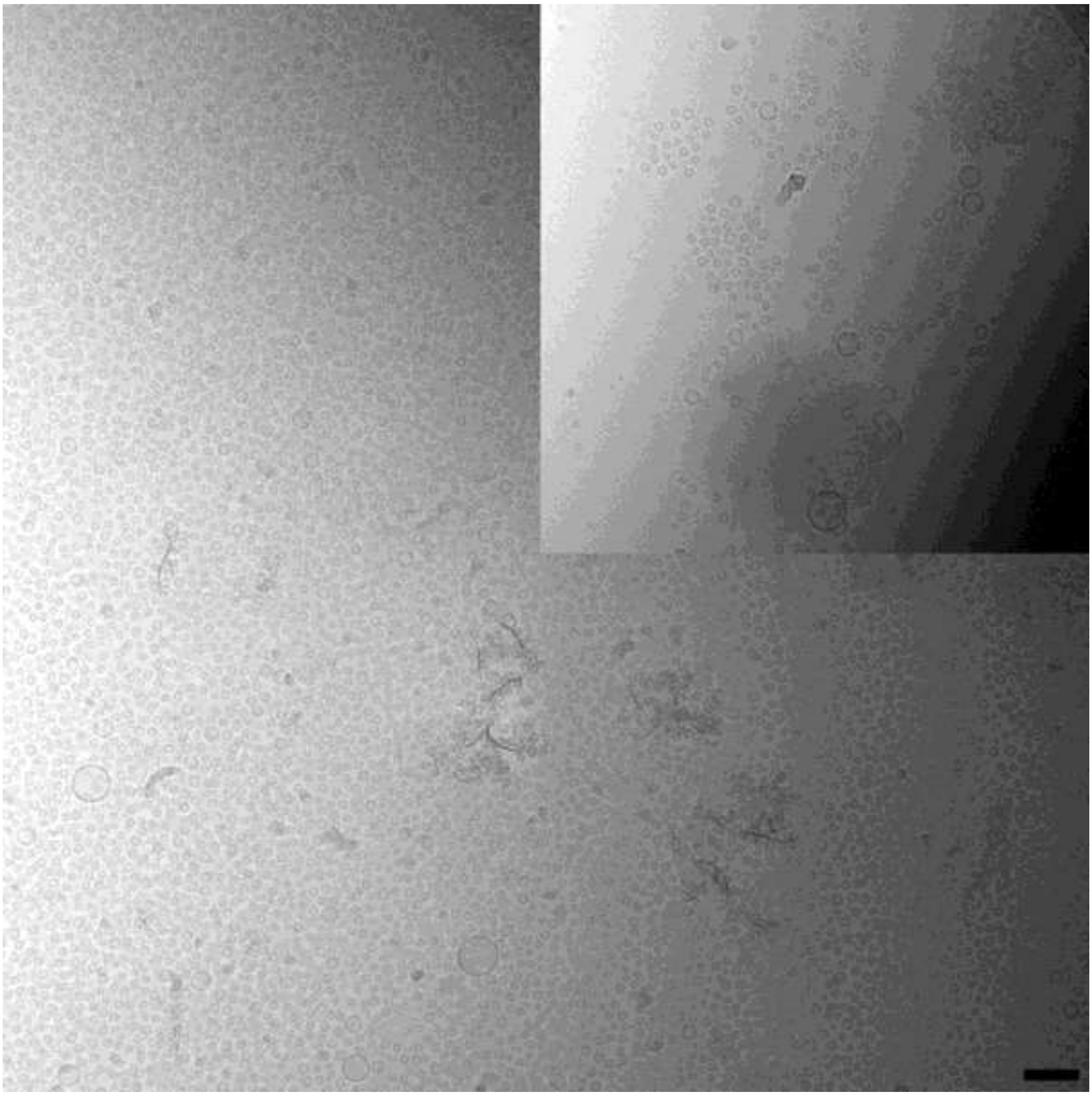

Figure S6: Cryo-TEM images for X=0.25, 5mM sample.]. The sample mainly consist of ultra-small small vesicles.

2.2 X=0.25, 20mM

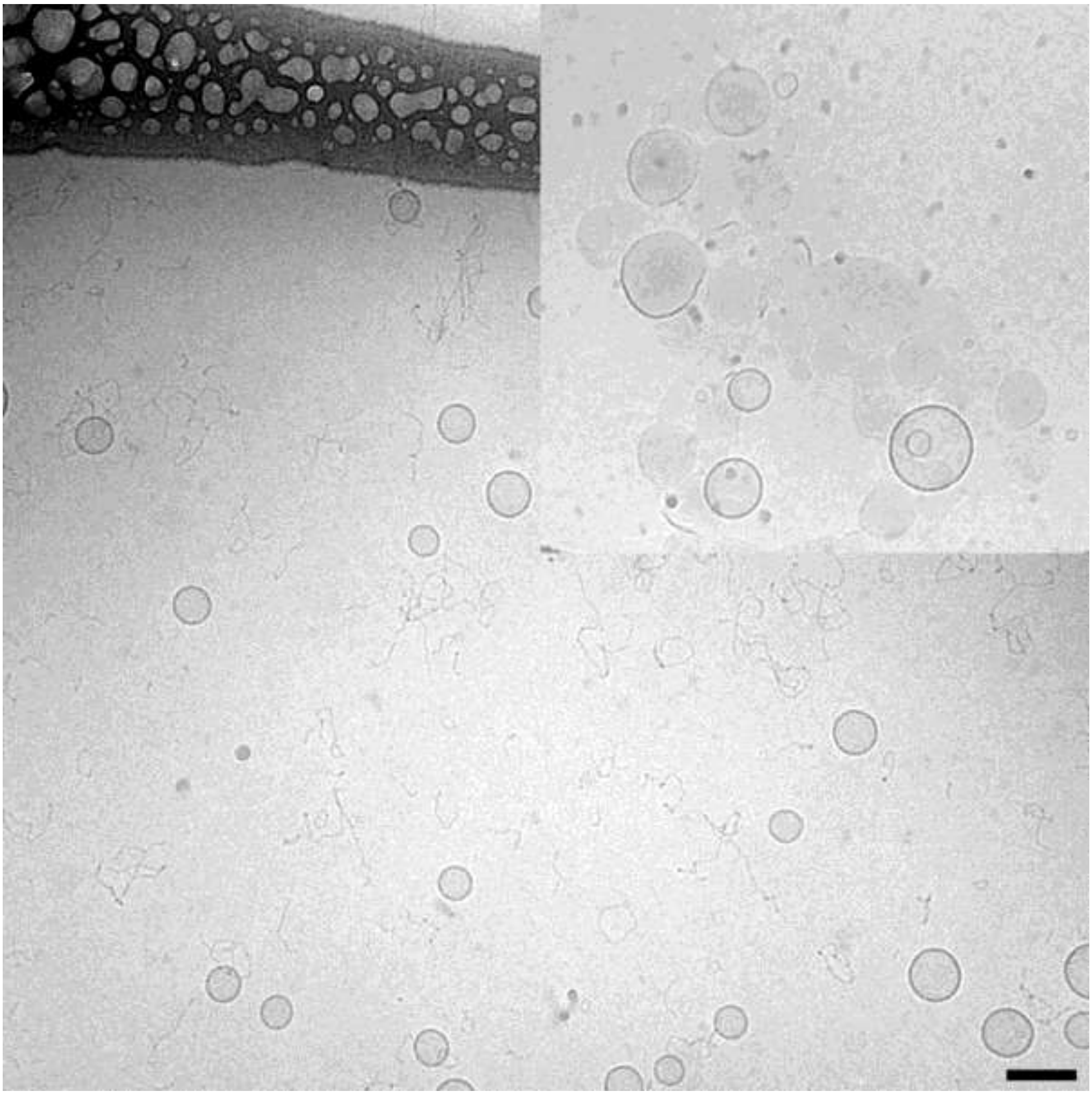

Figure S7: Cryo-TEM images for X=0.25, 20mM sample. Coexisting vesicle and wormlike micelles can be seen in the main image. The disks and vesicles are recognized in the inset plot..

2.3 Comparison between DOPC and DMPC vesicles

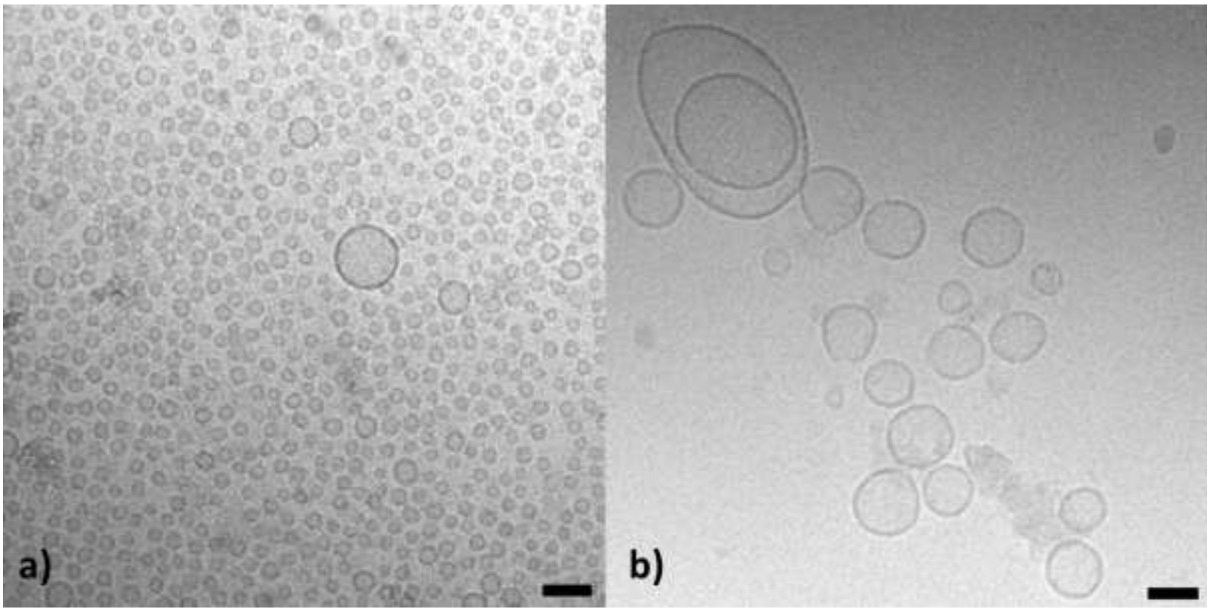

Figure S8: Comparison between the AMT-DOPC and AMT-DMPC vesicles. a) Cryo-TEM image of AMT-DOPC sample [X_{PL}=0.25, c_t= 5mM], see the manuscript text. b) Cryo-TEM image of AMT-DMPC sample [X_{PL}=0.25, c_t= 10mM. The vesicles are significantly bigger in size than the corresponding AMT-DOPC samples. The both scale bars are 50 nm.

3 Model employed in the least squares fitting data analysis

The differential scattering cross section as a function of scattering vector Q for a sample of non-interacting monodisperse micelles with concentration n (number of micelles per unit volume) can be written as follows [1]:

$$\frac{d\sigma(q)}{d\Omega} = n\Delta\rho^2 V^2 P(q) \tag{1}$$

where $\Delta\rho$ is the difference in scattering length density between aggregates and solvent and $P(q)$ is the form factor taking into account the geometrical shape of the aggregates.

Polydisperse rods. For elongated rodlike micelles, the form factor can be factorized in one contribution from the cross-section dimensions and one contribution for infinitely thin polydisperse rods, *i.e.*

$$P(q) = P_{ell}(q)P_{rod}(q) \tag{2}$$

The form factor $P_{cs}(q)$ for an homogeneous elliptical cross-section with half axes a and b, respectively, equals

$$P_{ell}(q,a,b) = \frac{2}{\pi}\int_0^{\pi/2} F^2\big(qr(a,b,\phi)\big)d\phi \tag{3}$$

$$F\big(qr(a,b,\phi)\big) = \frac{2B_1\big(qr(a,b,\phi)\big)}{qr(a,b,\phi)} \tag{4}$$

is the amplitude where $B_1(x)$ is the Bessel function of first order and

$$r(a,b,\phi) = \sqrt{a^2\sin^2\phi + b^2\cos^2\phi} \tag{5}$$

The form factor of infinitely thin rods that are polydisperse with respect to the length L of the rods equals

$$P_{rod}(q) = \frac{\int N_{rod}(L)L^2 S_{rod}(q,L)dL}{\int N_{rod}(L)L^2 dL} \tag{6}$$

where $N_{rod}(L)$ is the number distribution of micelles with respect to L and the form factor for an infinitely thin rod is given by [2]

$$S_{rod}(q,L) = 2\mathrm{Si}(qL) - 4\sin^2(qL/2)/(qL)^2 \tag{7}$$

and

$$\mathrm{Si}(x) = \int_0^x \frac{\sin t}{t}dt \tag{8}$$

In our data analysis we have assumed the number density of micelle lengths to follow a Schultz distribution in the entire range of aggregation numbers ($0 < N < \infty$), *i.e.*

$$N_{rod}(L) = \frac{L^z}{z!}\left(\frac{z+1}{\langle L\rangle_N}\right)^{z+1} e^{-L(z+1)/\langle L\rangle_N} \tag{9}$$

where $\langle L\rangle_N$ is the number-weighted average length of the micelles.

We have presented our results in Table S1 in terms of the volume-weighted average length $\langle L\rangle = (z + 2)/(z + 1)\times\langle L\rangle_N$, *i.e.* the mean value as calculated from the probability distribution of finding an aggregated surfactant in a micelle of length L, and the corresponding relative standard deviation $\sigma_L / \langle L\rangle = 1/\sqrt{z+2}$. Denoting the relative standard deviation for the number-weighted distribution in Eq. (9) p, $\langle L\rangle$ and $\sigma_L/\langle L\rangle$ for the volume-weighted distribution may be calculated from the relations

$$\langle L\rangle = (1 + p^2)\langle L\rangle_N \qquad (10)$$

and

$$\frac{\sigma_L}{\langle L\rangle} = \frac{p}{\sqrt{1+p^2}} \qquad (11)$$

Core-and-shell cross-section of rods. The SAXS data were fitted with a core-and-shell cross-section form factor. Hence, we can calculate the cross-section form factor from Eq. 3, using the following relation for the amplitude

$$F\big(qr(a,b,d,\phi)\big) = F_{core} + \varrho\frac{A_{shell}}{A_{core}}F_{shell} \qquad (12)$$

where

$$\varrho = \frac{\Delta\rho_{shell}}{\Delta\rho_{core}} \qquad (13)$$

and $\Delta\rho_{core}$ and $\Delta\rho_{shell}$ are the differences in scattering length densities between core and shell, respectively, and solvent. A_{core} is the area of an elliptical core cross-section with half axes a and b. $A_{shell} = A_{tot}$ ⍰ A_{core} is the area of the outer shell, where A_{tot} is the total area of the cross-section with half axes $a + d$ and $b + d$.

F_{core} is given by Eq. (4) and he corresponding quantity for the shell may be written as

$$F_{shell} = \frac{A_{tot}F\big(qr(a+d,b+d,\phi)\big) - A_{core}F\big(qr(a,b,\phi)\big)}{A_{shell}} \qquad (14)$$

where

$$r(a+d, b+d, \phi) = \sqrt{(a+d)^2\sin^2\phi + (b+d)^2\cos^2\phi} \qquad (15)$$

Bilayers vesicles and disks. In the coexistence region, the data could only be fitted with a model containing form factors of rodlike micelles, disk and vesicles. The corresponding form factor equals

$$P(q) = f_{disk}P_{bil}P_{disk} + f_{ves}P_{bil}P_{ves} + f_{mic}P_{cs}P_{rod} \qquad (16)$$

where f_{disk} and $f_{ves} = 1 - f_{disk}$ are the relative intensity-weighted fractions of bilayer disks and vesicles, respectively, and $f_{mic} = 1 - f_{disk} - f_{ves}$ is the intensity-weighted fraction of micelles. [3, 4]

The from factor of a homogeneous bilayer cross-section is

$$P_b(q\xi) = \left(\frac{sinq\xi}{q\xi}\right)^2 \qquad (17)$$

where we have assumed disks and vesicles to have identical half bilayer thickness ξ. The form factor of infinitely thin disks with disk radius R_d equals

$$P_{disk} = \frac{2[1-B_1(2qR_d)/qR_d]}{(qR_d)^2} \quad (18)$$

and $B_1(x)$ is the Bessel function of first order.

The form factor of polydisperse and infinitely thin vesicles with radius R can be written as

$$P_{ves} = \frac{\int N_v(R)R^4 S_v(qR)dR}{\int N_v(R)R^4(qR)} \quad (19)$$

where the form factor of an infinitely thin circular shell with radius R is defined as [1, 5]

$$S_v(qR) = \left(\frac{sinqR}{qR}\right)^2 \quad (20)$$

The vesicle size distribution is assumed to follow a Schultz distribution with respect to R^2, *i.e.*

$$N_v(R) = \frac{2R^{2z+1}}{z!}\left(\frac{z+1}{\langle R\rangle^2}\right)^{z+1} e^{-R^2(z+1)/\langle R\rangle^2} \quad (21)$$

where $\langle R\rangle$ is the average vesicle radius.

Least-squares model fitting. The reduced chi-squared used as a measure of the quality of the fits is defined as

$$\chi^2 = \frac{1}{N-M}\sum_{i=1}^{N}\left(\frac{I_{exp}(q_i)-I_{mod}(q_i)}{\sigma_i}\right)^2 \quad (22)$$

where $I_{exp}(q_i)$ and $I_{mod}(q_i)$ are the experimental and model intensities, respectively, at a scattering vector modulus q_i, σ_i is the statistical uncertainties on the data points, N is the total number of data points and M is the number of parameters optimized in the model fit.

4 Calculations of the Surfactant Mole Fraction in aggregates

The AMT free concentration above CMC is expected to depend on the composition in the aggregates. Assuming ideal mixing behaviour in the aggregates in our samples with comparatively high electrolyte concentrations, we may write $c_{PL}^{free} = x_{PL}CMC_{PL}$ and $c_{drug}^{free} = (1-x_{PL})CMC_{drug}$ for AMT and DOPC. Consequently, the concentrations of AMT and DOPC present in self-assembled aggregates (c_{agg}), as well as the aggregate mole fraction x_{PL}, may be calculated for a given total surfactant concentration $c_t = c_{agg} + c_{drug}^{free} + c_{PL}^{free}$ and bulk mole fraction of DOPC $X_{PL} = (c_{PL}^{free} + x_{PL}c_{agg})/c_t$. [6]

Taking into account that $c_{drug}^{free} \gg c_{PL}^{free}$, we obtain the following relation between mole fraction in solution and mole fraction in aggregates [7]

$$x_{PL} = X_{PL}\left(1 + \frac{c_{drug}^{free}}{c_{agg}}\right) \quad (23)$$

We have determined the CMC of AMT in 0.154 M NaCl solution to equal CMC_{drug}= 17 mM. The critical aggregate concentration of DOPC (CMC_{PL}) in 0.1 M salt have been reported to be in the range 1-10 µM [8].

References

1. Pedersen, J.S., *Analysis of small-angle scattering data from colloids and polymer solutions: modeling and least-squares fitting.* Advances in Colloid and Interface Science, 1997. **70**: p. 171-210.
2. Neugebauer, T., *Berechnung der lichtzerstreuung von fadenkettenlösungen.* Annalen der Physik, 1943. **434**(7-8): p. 509-533.
3. Bergström, M. and J.S. Pedersen, *A Small-Angle Neutron Scattering Study of Surfactant Aggregates Formed in Aqueous Mixtures of Sodium Dodecyl Sulfate and Didodecyldimethylammonium Bromide.* The Journal of Physical Chemistry B, 2000. **104**(17): p. 4155-4163.
4. Bergström, L.M., et al., *Spontaneous Transformations between Surfactant Bilayers of Different Topologies Observed in Mixtures of Sodium Octyl Sulfate and Hexadecyltrimethylammonium Bromide.* Langmuir, 2014. **30**(14): p. 3928-3938.
5. Bergström, M., et al., *Small-Angle Neutron Scattering (SANS) Study of Vesicles and Lamellar Sheets Formed from Mixtures of an Anionic and a Cationic Surfactant.* The Journal of Physical Chemistry B, 1999. **103**(45): p. 9888-9897.
6. Bergström, L.M. and M. Aratono, *Synergistic effects in mixtures of two identically charged ionic surfactants with different critical micelle concentrations.* Soft Matter, 2011. **7**(19): p. 8870-8879.
7. Forooqi Motlaq, V., et al., *Investigation of the enhanced ability of bile salt surfactants to solubilize phospholipid bilayers and form mixed micelles.* Soft Matter, 2021. **17**(33): p. 7769-7780.
8. Venkatesan, G.A., et al., *Evaporation-induced monolayer compression improves droplet interface bilayer formation using unsaturated lipids.* Biomicrofluidics, 2018. **12**(2): p. 024101.
